# A tale of two diseases

**DOI:** 10.1038/s41371-022-00798-3

**Published:** 2023-01-06

**Authors:** Stefanie Lip

**Affiliations:** BHF Glasgow Cardiovascular Research Centre, School of Cardiovascular and Metabolic Health, 126 University Place, University of Glasgow, G12 8TA Glasgow, UK

**Keywords:** Diseases, Hypertension

Despite its ‘silent killer’ sobriquet, the global efforts to tackle hypertension has been tepid at best. Its insidious impact on humanity over the last century is expected to last far into the future [1]. The initial blaze of research breakthroughs, resulting in the development of antihypertensive drugs supported by the largest clinical trial evidence-base to-date, has faltered. The current state of hypertension research is emblematic of the pondering pace of incremental science, additionally hamstrung by a myriad of diseases competing for finite research funding. In contrast, the response to the COVID-19 pandemic has been the antithesis of hypertension with unprecedented rapid global action bringing the devastating pandemic under control within 2 years—evidently, it was the worst of times and the best of times [2]. In this essay, I shall juxtapose the nearly 2 millennia of hypertension and 2 years of COVID-19 experiences, evoking hidden parallels between the two conditions to glean insights that would effectively direct the use of the £1 million grant to support hypertension research.

## It was the worst of times

Since the Yellow Emperor of China noted in 2600 BCE: *“[…] if too much salt is used for food, the pulse hardens*”, hypertension has grown into a global health crisis affecting over one billion people across the socio-economic spectrum, and directly causing 10.5 million deaths annually (~25000 deaths/day) [1]. Despite the availability of effective and inexpensive antihypertensive drugs only 1 in 5 patients have controlled hypertension and hypertension related cardiovascular death rates have increased over the last decade against previously declining death rates [3]. This highlights an urgent need to rethink strategies to minimise the global burden of hypertension

Early in the pandemic, hypertension emerged as a common comorbidity in patients hospitalized with COVID-19, accompanied by fears that hypertension may increase the risk of severe COVID-19 especially given the key role played by the ACE2 receptor in SARS-CoV-2 virus infection [4]. It became quickly evident that there was no direct interaction between hypertension and COVID-19, but this relief was short-lived. New outpatient visits for hypertension in the US decreased by 39% from 2020 compared with pre‐pandemic figures. The rise of virtual healthcare during the pandemic unintentionally excluded those with lower rates of healthcare literacy or with limited access to internet from chronic disease follow-up. The net detrimental impact on screening/control of cardiovascular risk factors during the pandemic exposed the lack of resilience of healthcare systems for non-communicable diseases, which appears to have caused more deaths than directly from COVID-19 [4]. Over the 2 years from 1^st^ January 2020, there were 6.2 million global deaths directly attributed to COVID-19, but during the same period the excess-mortality is estimated at 13.3–16.6 million(~20,000 deaths/day) [5] – this is strikingly similar to the pre-pandemic daily death toll from hypertension. Those at high cardiovascular disease (CVD) risk (elderly, minorities, socio-economically deprived) are disproportionately affected by COVID-19 [6] and are also dependent on long-term healthcare monitoring, suggesting CVD including hypertension are likely to be the main drivers of the indirect effects of COVID-19.

## It was the best of times

Over the last 55 years the pioneering Framingham Heart Study, the Multiple Risk Factor Intervention Trial, the landmark VA Cooperative Study, evidence synthesis by the Prospective Studies Collaboration, and innumerable randomized clinical trials have produced the largest evidence-based underpinning current hypertension management guidelines [7]. Between 1979 and 2007 the age-adjusted mortality rates for stroke and coronary heart disease declined by 57 and 63% respectively, revealing the transformative impact of hypertension research on health [8].

Early in the pandemic, the ACE2 receptor-mediated cell entry mechanism of SARS-CoV-2 triggered alarms about the use of RAAS inhibitors, but these were promptly allayed by observational studies [9]. More rigorous studies refuted the initial observations of a causal association between pre-existing hypertension and COVID-19 severity and mortality [10].

The value of national collaboration was emphasised by the Randomised Evaluation of COVID-19 Therapy (RECOVERY) trial [11], a large adaptive platform trial (multi-arm trial allowing for comparisons between multiple drugs with arms being dropped or added mid-trial) [12] evaluating interventions for hospitalised COVID-19 patients across 176 hospitals in the UK. This trial impressively recruited 11,841 patients in 4 months (March–June, 2020) to produce the first evidence-based treatment for severe COVID-19 - dexamethasone [11]. Furthermore, it conclusively debunked hydroxychloroquine for COVID-19. Collaborations also enabled the unprecedent pace of vaccine development leading to 23 vaccines against SARS-CoV-2 being approved globally (and >8 billion doses administered) with more in development [13].

## It was the age of foolishness

The numerous triumphs in hypertension and COVID-19 research have been accompanied by just as many missteps. Recent data shows declining blood pressure (BP) control rates, with the greatest deterioration seen in minority and low-income populations [14]. Between 2000 and 2018, US data show a consistent worsening of national mortality rates for all hypertension-related CVD [3]. Although home BP monitoring is the recommended reliable approach, a recent report from the Lancet Commission on Hypertension found that only 15% of the 3000 commercially available BP measuring devices are validated [15]. Physician inertia and inadequate pharmacological treatment continue to remain the key drivers of poor BP control rates. A recent report found that 40% of patients with uncontrolled hypertension were only on monotherapy [16]. Telemonitoring and virtual healthcare touted to hold great promise for hypertension management have been shown to exacerbate inequities owing to the lack of universal access to smartphones, computers, and internet access [17].

The medical research community’s response to COVID-19 has arguably been inefficient and wasteful, with an overwhelmingly large number of clinical trials being two-arm trials with redundant control groups, carried out with questionable methodological rigour, incorrect sample size estimates and failure to complete recruitment [18]. Furthermore lack of standardised operating procedures, harmonised endpoints, or robust data sharing mechanisms make it impossible to generalise conclusions through meta-analyses.

The inadequate representation of low- and middle-income countries (LMICs) in COVID-19 trials is representative of healthcare research in general. The astonishing success of vaccine roll-out has been undermined by the stark inequity it has exposed - on average 83% of the eligible populations in high-income countries have had at least one dose of the vaccine in contrast to 21% in LMICs [18].

## It was the age of wisdom

In hypertension, the importance of patient empowerment through education and engagement is evident by the widespread promotion and acceptance of home BP monitoring [19]. Team‐based care led by pharmacist and ancillary providers is actively being investigated as a means to decrease inequities in hypertension outcomes [20]. Notably, health promotion in black-owned barbershops resulted in a significant BP reduction when coupled with medication management by pharmacists [21].

COVID-19 dramatically and rapidly changed research culture by driving the use of platform trials and master protocols. Under a master protocol framework, platform trials establish a large trial network with a common infrastructure across multiple institutions that are able to join and leave over time. The WHO’s master protocol for COVID-19 (February 2020) outlined plans for clinical trial evaluation in hospitalised patients across multiple research teams leading to the early discovery of treatments (RECOVERY, SOLIDARITY trials) [11, 22].

## From the winter of despair to the spring of hope

This essay was stimulated by the challenge to support a hypertension research project with a £1 million grant. Given the parallels between the COVID-19 and hypertension, I shall apply the lens of COVID-19 to inform where to target this investment to maximise its value to hypertension research.

The ability to carry out randomised trials rapidly and adaptively alongside the ability to scale-up recruitment using digital tools turned the tide of COVID-19. Hence, I will focus on these two domains as high-priority areas for investment. I posit that transforming these two domains will result in the quickest and largest impact on patient care accompanied by a trickle-down effect on the basic science research by opening routes to clinical translation.

### Devices

The transformative power of digital technologies in healthcare is undeniable. Cuff-less devices utilise sensors to capture signals from ECG or photoplethysmogram to estimate BP with promising preliminary data on accuracy and reliability. The opportunities afforded by breaking away from traditional cuff-based measurement of BP include reducing the burden of clinic visits, more efficient and real-time remote monitoring, active/passive patient assessment, investigate traditional and novel endpoints captured in an automated fashion over extended periods. Furthermore, cost-effective devices with low-power requirements will enhance conduct of trials in traditionally poorly represented LMICs. Thus, evaluating and validating these devices is a key first step, but there is a lack of universally agreed standards for the validation of such technology [23]. The first £250 K investment will develop standards for validation of next-generation BP devices, conduct rapid and rigorous evaluation studies to accelerate these devices along the road to regulatory approval.

The availability of easy to use and informative mobile apps that interpret BP and advise patients and providers has the potential to improve health literacy, improve engagement with digital enabled trials and ultimately improve BP control in the individual. A survey of BP apps showed that only 4% of 180 apps available to measure BP had any involvement of medical experts in their development and none have obtained approval from either US or European regulators [24]. The American Heart Association found almost all smartphone BP apps to be erroneous [23, 24]. Additional efforts are required to improve and adapt these apps for those with cognitive deficits and visual or hearing impairments. The second tranche of investment (£250 K) will be to improve the usability of patient-facing mobile apps. This will bring together patients, physicians, nurses, and technologists to co-develop an app that will be fit for purpose, acceptable to the users, and supported by trial evidence.

### Improving patient recruitment and retention

The demographics that are particularly difficult to enrol in clinical trials are ethnic minorities, older adults, and children. For example, cardiology trials in the US have recruited only 2.5% of Black Americans [25], 40% of all cardiology clinical trials exclude older adults, and trials in children are challenging. Hypertension has no demographic predilection, and it behoves hypertension researchers to ensure fair representation in their studies. Once patients are recruited into a trial, the next obstacle to successful completion of a trial is patient retention. Concerns regarding randomization and assignment to a placebo arm, general lack of understanding of the clinical trial process, lack of renumeration are some of the reasons why trial participants fail to complete trials.

There is substantial evidence supporting the power of digital technology in clinical trial recruitment. But regulatory guidance is currently only available for digital informed consent. Hence the third tranche of investment (£250 K) will be to develop social media toolkits and communication strategies to help overcome barriers such as mistrust, access, and fear of human experimentation; raise awareness amongst investigators, sponsors, and ethics committees in order to facilitate wider use of these tools and result in quicker recruitment of representative and underserved populations; develop digital bolt-ons (to eCRF and other follow-up processes currently used in trials technologies) that allow more efficient participant outreach, minimising the burden of clinic visits, and incorporation of video and other visual formats to aid understanding and engagement. The value of generating results that demonstrate external validity cannot be overemphasised and this investment is critical to deliver this objective.

### Data privacy, security, and trust

Digital technology and remote monitoring heighten the need for security measures to protect against data breaches during collection, transmission, and/or storage of data. To this end, the FDA has embraced cybersecurity as a component of medical device certification. Blockchain is being investigated as a platform to overcome the barriers posed by the centralised system of conducting clinical trials, trust, and ownership of data. Blockchain is a transaction record that is immutably stored and linked up cryptographically, so that there is a single source of truth engendering trust. The fourth tranche of investment (£250 K) will be across a portfolio of measures to improve privacy and trust. For instance, evaluating blockchain-related technologies for trial integrity, decentralised trials, patient incentivisation, and exploring opportunities for patient ownership of data.

**Fig. 1:**
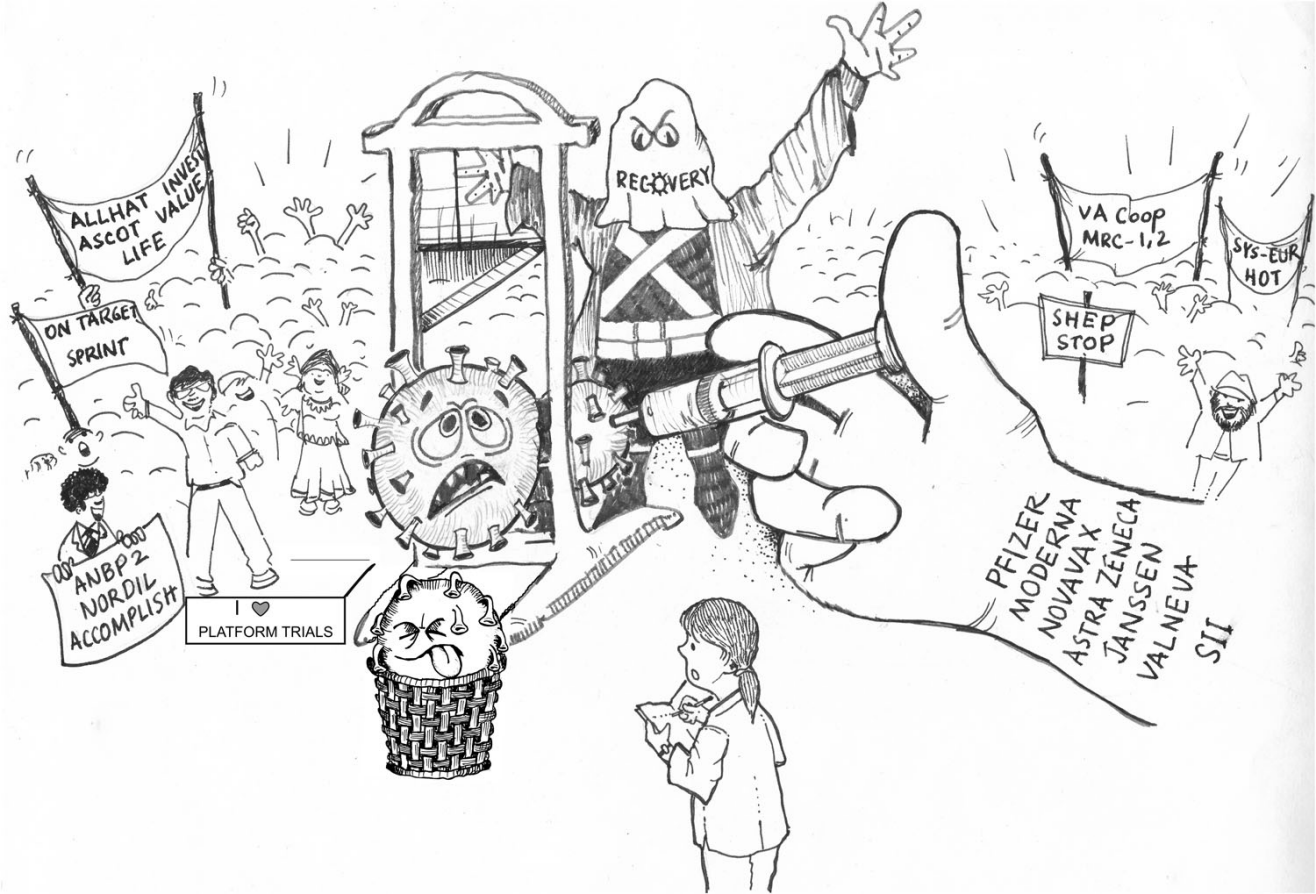
COVID-19 pandemic and hypertension. “It is a far, far better thing that I do, than I have ever done; it is a far, far better rest I go to than I have ever known.” Dr. Stefanie Lip draws inspiration from the sacrifices and scientific advances during the COVID-19 pandemic to envision how hypertension research can be transformed. Illustration by Dr. Kushal K Choudhuri.

In conclusion, the terrible cost of COVID-19 offers redemption for hypertension learning and adapting techniques that proved effective in bringing the pandemic to a halt. (Fig. 1)

**Fig. 2:**
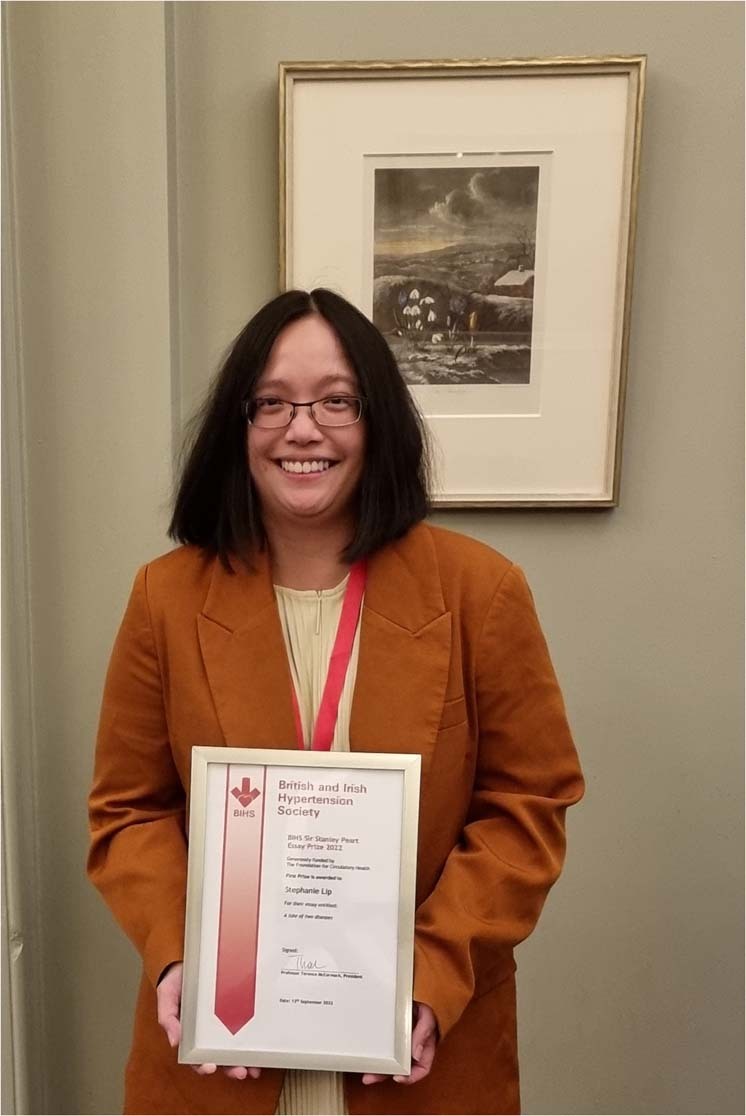
Dr. Stefanie Lip with the British and Irish Hypertension Society Sir Stanley Peart Essay prize.

This essay won the first place in the British and Irish Hypertension Society Sir Stanley Peart Essay competition. (Fig. 2)
